# Sizing Up the Uncultured Microbial Majority

**DOI:** 10.1128/mSystems.00185-18

**Published:** 2018-09-25

**Authors:** Laura A. Hug

**Affiliations:** a Department of Biology, University of Waterloo, Waterloo, Ontario, Canada

**Keywords:** amplicon sequencing, metagenomics, metatranscriptomics, microbial diversity

## Abstract

Predicting the total number of microbial cells on Earth and exploring the full diversity of life are fundamental research concepts that have undergone paradigm shifts in the genomic era. In this issue, Lloyd and colleagues (K. G. Lloyd, A. D. Steen, J. L. Ladau, J. Yin, and L. Crosby, mSystems 3:e00055-18, https://doi.org/10.1128/mSystems.00055-18, 2018) present results that combine these two concepts by estimating the total diversity of all cells from Earth’s environments. Leveraging publicly available amplicon, metagenomic, and metatranscriptomic datasets, they determined that nearly all environments are dominated by uncultured lineages, with the exception of humans and human-associated habitats. They define a new concept: phylogenetically diverse noncultured cells (PDNC). Unlike viable but nonculturable cells (VBNC), PDNC are microorganisms for which traditional isolation techniques may never succeed. Lloyd et al. estimate that the majority of microorganisms in Earth’s ecosystems may be PDNC and conclude that culture-independent methods combined with innovative culturing techniques may be required to understand the ecology and physiology of these abundant and divergent microorganisms.

## Abstract (precis)

Predicting the total number of microbial cells on Earth and exploring the full diversity of life are fundamental research concepts that have undergone paradigm shifts in the genomic era. In this issue, Lloyd and colleagues (K.

## COMMENTARY

Attempts to characterize microbial life on Earth typically fall into two research categories. Either they seek to quantify the total number of microbial cells on the planet (see, e.g., references 1, 2, and 3) or they strive to describe the entirety of microbial diversity from phylogenetic and taxonomic perspectives (4–6). New work from Lloyd and colleagues (7), published in this issue, attempts to connect these two categories by defining the diversity of all microbial cells from available sequence data derived from Earth’s diverse environments. Their results suggest that the majority of microorganisms detected within nearly all samples affiliate with divergent and uncultured lineages, despite these microbial cells being relatively abundant and likely important to ecosystem functioning within their respective source environments (7).

**FIG 1:**
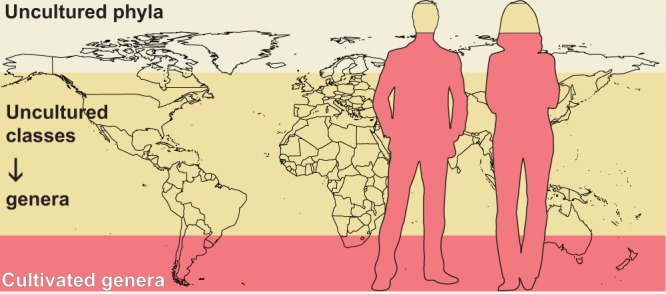
Graphical representation of data adapted from the work by Lloyd et al. (7) showing the proportion of uncultured cells from Earth’s habitats, with an inset demonstrating that culturability is highest for humans and human-associated environments. Based on the conservative simplification of a single 16S rRNA gene per genome, Lloyd and colleagues estimate that 19% of Earth’s microbial cells are from cultivated lineages (pink). The remaining 81% are cells from uncultured genera or higher levels of taxa, with 25% from uncultured phyla (cream). In contrast, microbial populations from humans are overwhelmingly from cultured genera, with only ∼1% of cells belonging to uncultured phyla. (Image generated from the R package “maps” and royalty-free clipart.)

Lloyd and colleagues mined 16S rRNA genes, metagenomes, and metatranscriptomes from publicly available databases. Simultaneously, they counted and classified microorganisms within the selected habitats by comparing environmentally derived 16S rRNA gene sequence reads to full-length reference sequences from cultivated microorganisms in the SILVA database (8, 9). Their analyses showed that the vast majority of Earth’s environments are dominated by taxa from uncultured lineages (Fig. 1). Only human and human-associated habitats (e.g., indoor and outdoor aerosols, drinking water, building materials) showed the reverse pattern, with the majority of detected microorganisms belonging to genera with cultured representatives (Fig. 1). It is striking that this global dominance of uncultured microbial taxa is consistent for systems that directly impact human activities, including livestock, agricultural soils, and engineered bioreactors. Microbial communities in systems with obvious economic importance remain uncultured to a similar extent as those in extreme environments such as hydrothermal vents. These findings highlight ongoing research bias toward human systems and human health in microbiology research but may also reflect the comparative ease of mimicking human-associated habitats in a laboratory.

The comparison between the amplicon sequencing and metagenomic data sets identified important differences between these techniques, as well as probable biases in our current understanding of microbial diversity. As expected, amplicon sequencing data underestimated the proportion of phylogenetically divergent microorganisms compared to total community sequencing methods, with approximately 35% of amplicon sequences classified to the genus level with cultured members compared to only 15% of metagenome-derived 16S rRNA genes. This discrepancy is likely due to traditional primer design approaches favoring cultured microorganisms in the resulting amplicon sequence data. Archaea contributed a higher proportion of sequences from highly divergent lineages than bacteria in both amplicon and meta-omic data sets. From the 16S rRNA gene amplicon data, 44% of archaeal sequences were assigned to uncultured phyla compared to 17% of bacterial sequences. Our understanding of the domain Archaea is undergoing rapid expansion as culture-independent methods allow access to new environments and low-abundance microorganisms (10, 11). The results reported by Lloyd et al. indicate that the lower diversity of sequences from archaea than from bacteria in current genome databases may be, in part, a consequence of sampling bias (4).

Lloyd and colleagues made data handling decisions that impact their estimates. First, they used 16S rRNA gene sequence identity thresholds of 86% and higher to define phylum boundaries (12), which is a conservative choice compared to the thresholds of 75% to 83% and higher that have recently, and historically, been used for defining novel phyla identified through culture-independent methods (13–15). Were a lower threshold for phyla used (e.g., 80%), the proportion of detected taxa assigned to novel uncultured phyla might be much lower. This becomes a question of semantics, however, as these microorganisms would still be highly divergent and uncultured even if placed within a phylum with distantly related cultured relatives. Second, metatranscriptomic data are used to provide estimates of microbial abundance, despite the rRNA subtraction methods undoubtedly changing the abundance distribution of the organisms present (see, e.g., reference 16). Third, read depth is used to provide estimates of microbial abundance from metagenomes. This is problematic because unassembled reads of 16S rRNA genes can map spuriously to conserved regions of the 16S rRNA gene on assembled scaffolds. Most 16S rRNA genes in assembled metagenomes are on short scaffolds, with insufficient additional genomic information for an accurate average read depth to be calculated. They do not overstep these limitations in their analyses. Instead, they apply metatranscriptomic data primarily to confirm microbial viability in the environments surveyed, and they provide read depth-based estimates of abundance side by side with the count-based data for metagenomes. With these concerns in mind, their estimates based on metagenome-derived 16S rRNA gene counts likely suffer the least bias. From all analyses, it is clear that the dominance of abundant and highly divergent microbial cells from the majority of Earth’s environments is a robust finding regardless of the precise taxonomic rankings or exact numbers.

The key innovative concept introduced in this paper addresses the issue of culturability. A meta-analysis of cultivation trials from diverse environments yielded a median of 0.5% cultured cells—a new lower bound that updates the oft-cited 1% associated with the “Great Plate Count Anomaly” (17). Lloyd et al. predict that innovations in culture techniques will be successful for cultivation of viable but nonculturable cells (VBNC) (18). The hypothesis proposed by Lloyd et al. is that new cultivation methods do not succeed for the vast majority of microbial representatives from more divergent taxa. These divergent lineages may comprise as much as 95% of taxa in some environments and may require nonaxenic growth conditions to survive in a laboratory environment. Lloyd et al. coin the name and define the concept of phylogenetically divergent noncultured cells (PDNC), characterized here as representatives from the order level or higher with no cultured representatives. The PDNC represent lineages whose metabolisms may preclude growth as pure cultures, including obligate syntrophs, extreme oligotrophs, and cells with very low growth rates. In contrast to VBNC, which are dormant cells awaiting the correct conditions for growth, the PDNC may require fundamentally different techniques to be brought into culture. Lloyd et al. suggest the PDNC and VBNC can be separated by taxonomic novelty, with organisms novel at the order level or higher belonging to the PDNC. It seems unlikely this suggested boundary will hold in the future, as several studies have already reported cultivation of highly novel organisms using relatively routine methods (see, e.g., references 19 and 20). PDNC will instead be applied to those cells resisting cultivation efforts or requiring innovative new protocols to be brought into the laboratory environment.

The PDNC is an important concept with respect to analyses of environmental microbial communities, and members of the PDNC represent the current grand challenge for cultivation assays. Lloyd and colleagues conclude that culture-independent techniques and mixed enrichment cultures may be the best avenues for studying the physiology and ecology of these abundant and phylogenetically divergent cells. Their report helps refocus attention on the challenge of cultivating microorganisms because, as they state, “uncultured microbes, often from deeply phylogenetically divergent groups, dominate nonhuman environments on Earth, and their undiscovered physiologies may matter for Earth systems."
